# Surface guided imaging during stereotactic radiosurgery with automated delivery

**DOI:** 10.1002/acm2.13066

**Published:** 2020-10-23

**Authors:** Elizabeth L. Covington, Dennis N. Stanley, John B. Fiveash, Evan M. Thomas, Samuel R. Marcrom, Marcus Bredel, Christopher D. Willey, Kristen O. Riley, Richard A. Popple

**Affiliations:** ^1^ Department of Radiation Oncology University of Alabama – Birmingham Birmingham AL USA; ^2^ Department of Neurosurgery University of Alabama – Birmingham Birmingham AL USA

**Keywords:** intrafraction motion, optical surface imaging, SGRT, stereotactic radiosurgery, surface guided radiotherapy

## Abstract

**Purpose:**

To report on the use of surface guided imaging during frameless intracranial stereotactic radiotherapy with automated delivery via HyperArc^TM^ (Varian Medical Systems, Palo Alto, CA).

**Methods:**

All patients received intracranial radiotherapy with HyperArc^TM^ and were monitored for intrafraction motion by the AlignRT® (VisionRT, London, UK) surface imaging (SI) system. Immobilization was with the Encompass^TM^ (Qfix, Avondale, PA) aquaplast mask device. AlignRT® log files were correlated with trajectory log files to correlate treatment parameters with SI reported offsets. SI reported offsets were correlated with gantry angle and analyzed for performance issues at non‐zero couch angles and during camera‐pod blockage during gantry motion. Demographics in the treatment management system were used to identify race and determine if differences in SI reported offsets are due to skin tone settings.

**Results:**

A total of 981 fractions were monitored over 14 months and 819 were analyzed. The median AlignRT® reported motion from beginning to the end of treatment was 0.24 mm. The median offset before beam on at non‐zero couch angles was 0.55 mm. During gantry motion when camera pods are blocked, the median magnitude was below 1 mm. Median magnitude of offsets at non‐zero couch angles was not found to be significantly different for patients stratified by race.

**Conclusions:**

Surface image guidance is a viable alternative to scheduled mid‐treatment imaging for monitoring intrafraction motion during stereotactic radiosurgery with automated delivery.

## 1 INTRODUCTION

HyperArc^TM^ is comprised of both a treatment planning and automated delivery component for single isocenter, volumetric modulated arc‐therapy (VMAT) for intracranial stereotactic radiosurgery (SRS) including treatment for multiple targets simultaneously. The treatment planning system (TPS) component within Eclipse (Varian Medical Systems, Palo Alto, CA) allows for high‐quality SRT planning with limited dosimetry experience and a more streamlined workflow. Pre‐defined angles are selected for treatment and isocenter selection is used to ensure no collision risk during treatment. Patient immobilization must use the Encompass SRS Immobilization System (Qfix, Avondale, PA) for proper collision mapping and radiosurgery specific normal tissue objective. To monitor for intrafraction motion, HyperArc^TM^ allows MV imaging at designated waypoints during treatment. Stopping the automated delivery to acquire and review images increases the length of the treatment and may increase the chance for patients to move. An alternative to mid‐treatment imaging is surface imaging, which uses optical tracking of the patient's surface to monitor for intrafraction motion.

Recently, with the technological advancements in optical reconstruction and projection techniques, surface imaging has become increasingly more popular as a non‐invasive, non‐radiographic form of image guidance. Surface imaging (SI) systems use a combination of real‐time optical and laser‐based imaging techniques that have been shown to properly position patients,^1^, ^2^ accurately monitor, and quantify movement throughout the entirety of treatment,^3^, ^4^ and provide an accurate and reproducible respiratory surrogate for gating‐based deliveries.^5^, ^6^, ^7^, ^8^, ^9^, ^10^ The ability of SI systems to non‐radiographically collect a live surface image, determine positional correction vectors needed to match image to a predefined reference image, and monitor sub‐millimeter movements have made it a successful component of SRT where small targets and small margins are ever‐present important considerations. Surface imaging has seen a dramatic increase in clinical prevalence in radiotherapy clinics around the world. While SI has previously been evaluated for accuracy and clinical efficiency in traditional SRT,^11^, ^12^, ^13^, ^14^, ^15^ the evaluation of SI in conjunction with a 4pi based, automatic delivery has yet to be evaluated for clinical efficacy.

This study presents the largest cohort to date of patient data captured via surface guided imaging during SRT delivered with HyperArc^TM^. In this study, we examine the magnitude of translational intrafraction motion from beginning to the end of treatment, the magnitude of SI reported offsets at non‐zero couch angles, the impact of gantry motion on performance with respect to camera blockage, and the effect of skin tone on reported offset.

## 2 MATERIALS AND METHODS

All treatments were performed on an Edge linear accelerator (Varian Medical Systems, Palo Alto, CA) equipped with HyperArc^TM^. Surface imaging was performed with AlignRT®, also known as the Optical Surface Monitor System (Varian Medical Systems, Palo Alto, CA). In this study, all treatments were monitored with AlignRT® v5.1 which includes Advanced Camera Optimization (ACO). We have previously reported on system performance before and after the utilization of ACO.^16^

HyperArc^TM^ requires the use of the Encompass mask; therefore, all patients in this study were immobilized with the Encompass system which includes both a posterior molded head support and anterior open view mask compatible with surface imaging. The open‐face portion of the mask was wired during simulation to enable viewing of the open‐face region within the treatment planning system. The open‐face region was then contoured, the eye regions removed, and the resulting structure was exported to AlignRT® to be used as the region of interest (ROI) for SGRT. Prior to treatment, therapist turn on the projectors (i.e., start monitoring) for a minimum of 10 min to allow the cameras to reach thermal equilibrium. AlignRT requires that the user select the skin tone setting per patient. Skin tone setting is selected per the discrection of the user based on visual inspection of the patient. During treatment, each patient underwent radiographic imaging with both kV orthogonal imaging and cone‐beam CT (CBCT).

After radiographic alignment, a reference surface was captured in AlignRT®, and treatment was initiated. While HyperArc^TM^ has optional MV waypoints to monitor for intrafraction motion, these were not utilized in favor of monitoring with SI. SI reported offsets, called Real Time Deltas (RTDs) in AlignRT®, were monitored during treatment for patient motion. If the magnitude (MAG) of translational RTDs exceeded patient‐specific thresholds (typically 1 mm), the patient was returned to the reference position at couch zero. This was done by manually stopping the beam and not by use of gating thresholds and beam holds. If the MAG at couch zero was under the threshold, treatment was resumed. If RTDs remained above the threshold at the reference position, radiographic imaging was performed, shifts performed, and treatment was resumed.

Log files from AlignRT® continuously record RTDs from the patient’s reference position throughout treatment. SI system logs were correlated with information from the ARIA database (Varian Medical Systems) and linear accelerator trajectory log files. The SI log files and trajectory log files were synchronized using the initial beam‐on flag in each file.This enabled syncing RTDs with gantry angle toassess changes in RTDs with respect to the camera pod blockage by the gantry. The left and right camera pods were assumed to be at least partially blocked by the gantry at angles 303° ± 15° and 57° ± 15°.

The user selected skin tone setting is not documented in the SI log file; therefore, demographics in ARIA were used to identify the race of each patient as a surrogate for skin tone. Patients were classified in the following groups per ARIA: White, Black, or not‐specified (NS). Spot checks of skin tone settings were performed and confirmed that skin tone and race were correlated. This was done to study the impacts of SI skin tone settings on performance. We compared the magnitude of RTDs between the three groups to determine if there were differences due to suboptimal camera exposure settings. RTDs before beam on at non‐zero couch angle and the end of treatment were evaluated for the three patient cohorts.

Over 14 months, 981 fractions from 324 patients of SRT were analyzed. The average treatment time, defined as the interval from first beam‐on to the final beam‐off, was 2.85 min (range 1.62–7.95 min). Of the 981 fractions observed, 83 were omitted due to the inability to locate the corresponding trajectory log file. Twenty‐one additional fractions were omitted due to abnormal termination of treatment which caused a discontinuity in SI log file analysis. Twenty‐five were omitted due to a discordance between the SGRT and trajectory log beam on flags of >3 s. Thirty‐two (3.8%) fractions contained mid‐treatment imaging due to SI reported patient motion and were omitted due to discontinuity in RTDs due to new reference surface capture. Note that some fractions had multiple reasons for omission which left 844 fractions from 281 patients for analysis.

## 3 RESULTS

**Fig. 1:**
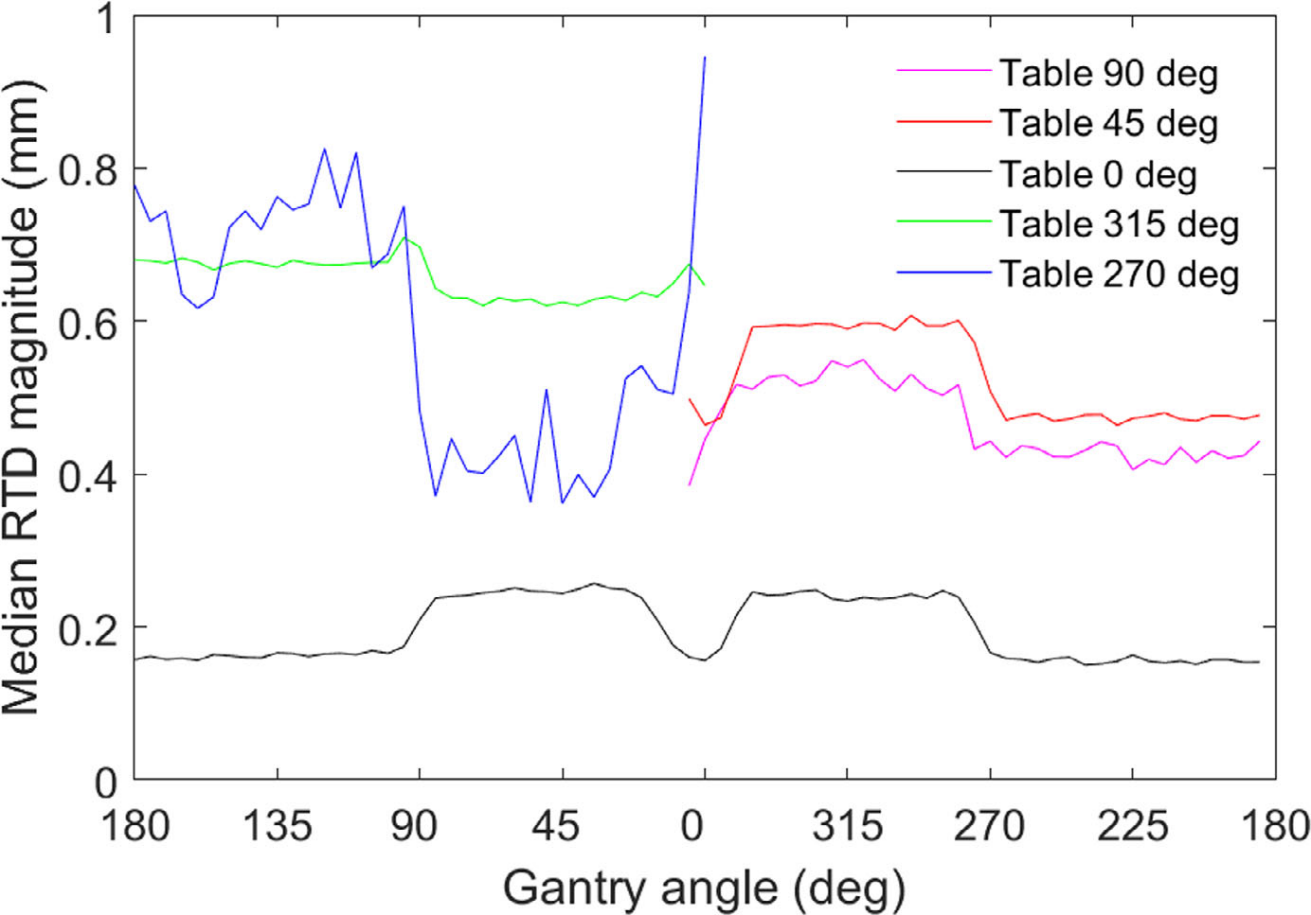
Median magnitude of surface imaging reported offsets, or Real Time Delta (RTD), versus couch angle for 819 fractions of SRT delivered via HyperArc^TM^.

**Fig. 2:**
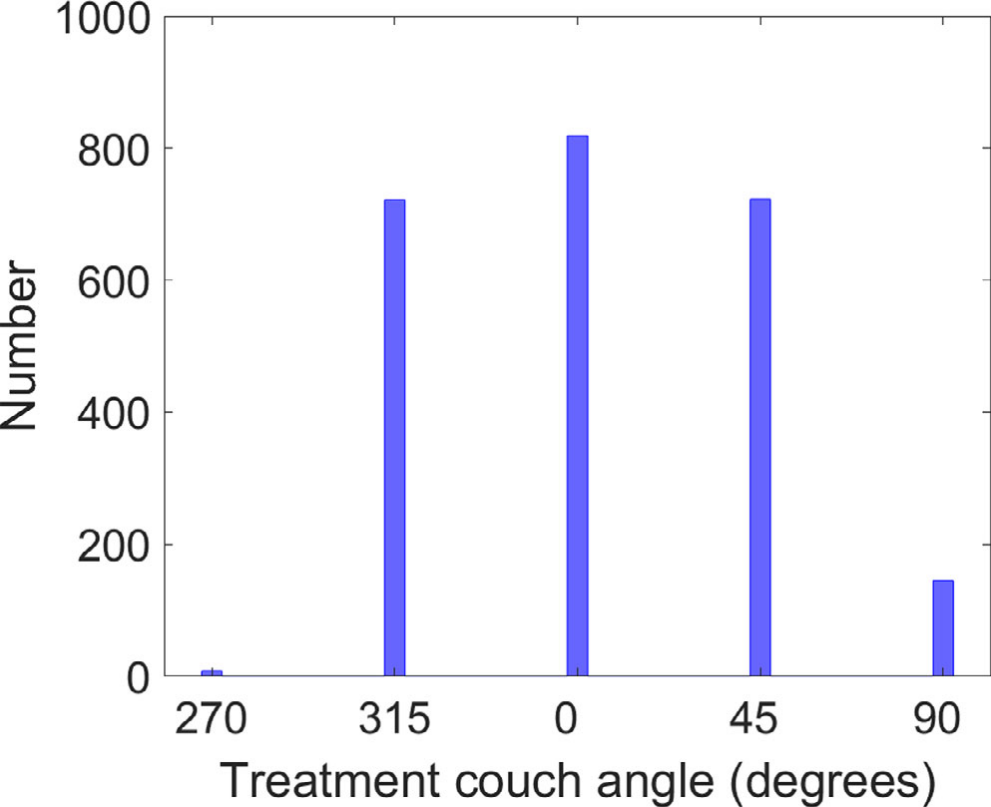
Number of clinically utilized couch angle in HyperArc^TM^ treatments for 819 fractions of SRT.

Table 1 shows the MAG for each group at the designated time‐points evaluated. The difference between Black and White patients was not found to be statistically significant (*P* = 0.127) using a Wilcoxon rank‐sum test. Statistical analysis was not performed for the NS group due to the limited number of patients. Figure 1 shows the median RTD magnitude versus gantry angle as determined via trajectory logs for all 819 fractions at the clinically utilized couch angles for HyperArc^TM^. The frequency of utilized couch angles is shown in Fig. 2. Table 2 shows the median and interquartile range (IQR) of translational offsets before beam on at non‐zero couch angles compared to the values at couch zero at the end of treatment for the 819 analyzable fractions.

**Table 1:** Median magnitude (MAG) of translational offsets reported via surface imaging during stereotactic radiosurgery delivered via HyperArc^TM^.

	Number of patients (%)	Number of fractions (%)	Median MAG (mm)
End of treatment	281	819	0.24
Before beam‐on at non‐zero couch angles	281	819	0.55
▪ White	207 (73.7%)	597 (72.9%)	0.55
▪ Black	59 (21.0%)	193 (23.6%)	0.53
▪ Race not specified	15 (5.3%)	29 (3.5%)	0.66

**Table 2:** Median and interquartile range (IQR) of translational offsets reported via surface imaging during stereotactic radiosurgery delivered via HyperArc^TM^ before beam on at non‐zero couch angles and at the end of treatment.

Couch angle (°)	Vertical (IQR) (mm)	Longitudinal (IQR) (mm)	Lateral (IQR) (mm)
270	−0.23 (0.11)	−0.64 (0.61)	0.10 (0.27)
225	−0.11 (0.20)	−0.41 (0.51)	−0.36 (0.29)
135	−0.08 (0.20)	−0.20 (0.48)	−0.21 (0.25)
90	−0.17 (0.27)	−0.05 (0.47)	−0.10 (0.26)
0° (End of treatment)	0.03 (0.15)	−0.03 (0.32)	0.00 (0.14)

Fractions with mid‐treatment imaging were omitted from the analysis in Table 1 due to multiple reference captures that prevent the analysis of RTDs from the beginning to the end of treatment. Shifts from radiographic imaging were analyzed for the 32 fractions, and 13 (40.6%) were found to be a false positive. False positives are defined as an SI reported MAG exceeding 1 mm but CBCT shifts had a magnitude <0.5 mm. The remaining 19 fractions (59.4%) were found to have CBCT confirmed patient motion with a median magnitude of 0.97 mm (range 0.51–2.76 mm).

## 4 DISCUSSION

The focus of this study was to analyze SI reported offsets with automated delivery; therefore, SI logs with a single reference capture were required. Patients with multiple reference captures were excluded from the analysis due to a discontinuity in SI logs which prevented comparison of patient position from the initial reference surface capture to the end of treatment. Per our previously reported workflow,^16^ CBCT is recommended to confirm patient movement due to false positives of intrafraction motion from the SI system at non‐zero couch angles. Of the 32 patients with mid‐treatment imaging, 41% were determined to be false positives; therefore, CBCT remains the standard for verifying intrafraction motion and patient alignment.

Not all stereotactic radiotherapy patients are treated with HyperArc^TM^. This study excludes functional SRT patients (trigeminal neuralgia, essential tremor, etc.) since they are treated with the virtual cone technique developed at our institution.^17^ Excluding virtual cone treatments, 98.7% of SRT plans were treated with HyperArc^TM^; therefore, only four eligible patients were not treated with HyperArc^TM^ during the period of this study. Three were due to not being simulated in the Encompass mask, and one was ineligible due to the lesion being located outside of the collision‐free zone required for HyperArc^TM^ delivery.

AlignRT® enables setting patient‐specific thresholds for reported out of tolerance RTDs or gating the beam. Our clinic does not enable gating via SI, rather RTDs are monitored by the treatment team, comprising a radiation oncologist, neurosurgeon, physicist, and therapists. If intrafraction motion tolerances are exceeded, the beam is manually stopped. The tolerance for returning to the reference position is typically 1 mm but could be modified for patient‐specific attributes (e.g., target proximity to critical organs at risk). In a previous study with traditionally delivered SRT, 4.3% of patients had CBCT confirmed patient movement while this study reports 2.2%.^16^ This reduction in motion could be attributed to the decrease in mean treatment from 4.84 min (range 1.15–37.27 min) to 2.86 min (range 1.62–7.95 min).

Anecdotal reports for SI system users indicated performance issues that may be attributed to patient‐specific characteristics. Skin tone was chosen as an attribute to investigate due to camera performance being dependent on proper exposure settings. Note that AlignRT® requires the user to specify the skin tone of the patient. During the time of this study, the software had three choices for skin‐tone: fair, mid, and dark. The current version of AlignRT® Advance v6.2 (Vision RT, London, UK) now has five available settings for skin‐tone. This choice sets the exposure times of the cameras and are aimed to improve the variations that can affect the performance of RTDs due to skin tone. Darker skin tones will absorb more of the projected speckle pattern; therefore, longer exposure time is needed to allow sufficient light to reach the sensor. If the optimal skin tone is not selected, there is a risk of under or over‐exposing the image resulting in inferior surface tracking. A hypothesis was that relying on users to select skin tone settings could result in suboptimal performance; however, our data did not find a difference between the RTDs before beam‐on at non‐zero couch angles for the two largest cohorts of patients studied.

Similar to a previous study, RTDs at non‐zero couch angles were larger than RTDs observed at the end of treatment, indicating SI system performance is still sub‐optimal at non‐zero couch angles. Additionally, the largest component of the translation magnitude continues to be from offsets reported in the longitudinal direction as shown in Table 2 Minimal offsets in the vertical direction are also consistent with previously reported aggregate data.^16^ Larger RTDs were noted on the 270° side of the treatment couch suggesting differences that may be attributed to camera pod geometry or an individual camera's performance. A limitation of this study is that the aggregate data was collected on a single SGRT system; therefore, additional studies are needed across multiple systems to confirm the trends reported in this study.

HyperArc^TM^ users have the option of utilizing mid‐treatment imaging via the electronic portal imaging device (EPID) to take MV images at designated time points. This is referred to as waypoint imaging and is scheduled to be taken before beam‐on at non‐zero couch angles. While waypoint imaging can be utilized by clinics with and without surface imaging, its utility is restricted due to the MV images being restricted to AP/PA imaging; therefore, vertical offsets are not reported. Waypoint imaging is also vulnerable to variability between users and sensitive to the region of interest set if auto‐matching is utilized.^18^ Surface imaging provides a more efficient workflow for reported intrafraction motion in all translational and rotational directions with minimal false positives.

Another benefit of SI monitoring is continuous logging of RTDs throughout treatment rather than at specified radiographic imaging time‐points. This allows for aggregate data analysis of camera performance at all couch and gantry angles utilized clinically. Analysis of SI system logs with trajectory logs allows correlating RTDs with the corresponding gantry angle. Figure 1 shows how the camera systems behave when the camera pods are blocked by the gantry. Changes in RTDs are visible when the camera pods are blocked by the gantry, but aggregate data shows the increases are sub‐millimetric. While the reported offsets slightly increase during camera pod blockage for most couch angles, a slight decrease is seen for couch 315°. We believe these results are likely dependent on the details of the camera pod geometry and camera configuration and may not indicate performance for other clinics.

## 5 CONCLUSIONS

Surface image guidance is a viable alternative to scheduled mid‐treatment x‐ray imaging for HyperArc^TM^ patients. Surface image offers sub‐millimeter accuracy with real‐time measurements and no loss of treatment efficiency. SI offsets observed during HyperArc^TM^ are on the order of offsets observed during traditionally delivered SRT. Analysis in conjuction with linear accelerator trajectory logs can be used to assess system performance when camera pods are blocked by the gantry and enable an aggregate analysis of patient‐reported offsets during automated delivery.

## AUTHOR CONTRIBUTIONS

Elizabeth L. Covington made substantial contributions to the conception and design of the work; the acquisition, analysis, and interpretation of data for the work; drafting the work and revising it critically for important intellectual content; gave final approval of the version to be published; and agrees to be accountable for all aspects of the work in ensuring that questions related to the accuracy or integrity of any part of the work are appropriately investigated and resolved.

Dennis N. Stanley made substantial contributions to the interpretation of data for the work; revising it critically for important intellectual content; gave final approval of the version to be published; and agrees to be accountable for all aspects of the work in ensuring that questions related to the accuracy or integrity of any part of the work are appropriately investigated and resolved.

John B. Fiveash made substantial contributions to the interpretation of data for the work; revising it critically for important intellectual content; gave final approval of the version to be published; and agrees to be accountable for all aspects of the work in ensuring that questions related to the accuracy or integrity of any part of the work are appropriately investigated and resolved.

Evan M. Thomas made substantial contributions to the interpretation of data for the work; revising it critically for important intellectual content; gave final approval of the version to be published; and agrees to be accountable for all aspects of the work in ensuring that questions related to the accuracy or integrity of any part of the work are appropriately investigated and resolved.

Samuel R. Marcrom made substantial contributions to the interpretation of data for the work; revising it critically for important intellectual content; gave final approval of the version to be published; and agrees to be accountable for all aspects of the work in ensuring that questions related to the accuracy or integrity of any part of the work are appropriately investigated and resolved.

Marcus Bredel made substantial contributions to the interpretation of data for the work; revising it critically for important intellectual content; gave final approval of the version to be published; and agrees to be accountable for all aspects of the work in ensuring that questions related to the accuracy or integrity of any part of the work are appropriately investigated and resolved.

Christopher D. Willey made substantial contributions to the interpretation of data for the work; revising it critically for important intellectual content; gave final approval of the version to be published; and agrees to be accountable for all aspects of the work in ensuring that questions related to the accuracy or integrity of any part of the work are appropriately investigated and resolved.

Kristen O. Riley made substantial contributions to the interpretation of data for the work; revising it critically for important intellectual content; gave final approval of the version to be published; and agrees to be accountable for all aspects of the work in ensuring that questions related to the accuracy or integrity of any part of the work are appropriately investigated and resolved.

Richard A. Popple made substantial contributions to the conception and design of the work; the acquisition, analysis, and interpretation of data for the work; revising it critically for important intellectual content; gave final approval of the version to be published; and agrees to be accountable for all aspects of the work in ensuring that questions related to the accuracy or integrity of any part of the work are appropriately investigated and resolved.

## CONFLICT OF INTEREST

Elizabeth L. Covington has received honorarium and travel assistance from VisionRT. Evan M. Thomas, Richard A. Popple, and John B. Fiveash have received honoraria from Varian Medical Systems which are outside of the scope of this article. University of Alabama‐Birmingham has educational and research grants from Varian Medical Systems.
